# Assessing the Cost of Global Biodiversity and Conservation Knowledge

**DOI:** 10.1371/journal.pone.0160640

**Published:** 2016-08-16

**Authors:** Diego Juffe-Bignoli, Thomas M. Brooks, Stuart H. M. Butchart, Richard B. Jenkins, Kaia Boe, Michael Hoffmann, Ariadne Angulo, Steve Bachman, Monika Böhm, Neil Brummitt, Kent E. Carpenter, Pat J. Comer, Neil Cox, Annabelle Cuttelod, William R. T. Darwall, Moreno Di Marco, Lincoln D. C. Fishpool, Bárbara Goettsch, Melanie Heath, Craig Hilton-Taylor, Jon Hutton, Tim Johnson, Ackbar Joolia, David A. Keith, Penny F. Langhammer, Jennifer Luedtke, Eimear Nic Lughadha, Maiko Lutz, Ian May, Rebecca M. Miller, María A. Oliveira-Miranda, Mike Parr, Caroline M. Pollock, Gina Ralph, Jon Paul Rodríguez, Carlo Rondinini, Jane Smart, Simon Stuart, Andy Symes, Andrew W. Tordoff, Stephen Woodley, Bruce Young, Naomi Kingston

**Affiliations:** 1 United Nations Environment Programme, World Conservation Monitoring Centre (UNEP-WCMC), 219 Huntingdon Road, CB3 0DL Cambridge, United Kingdom; 2 International Union for Conservation of Nature (IUCN), 28 rue Mauverney, 1196 Gland, Switzerland; 3 World Agroforestry Center (ICRAF), University of the Philippines Los Baños, Laguna 4031, Philippines; 4 Institute for Marine and Antarctic Studies, University of Tasmania, Hobart TAS 7001, Australia; 5 BirdLife International, David Attenborough Building, Pembroke Street, Cambridge CB2 3QZ, United Kingdom; 6 Department of Zoology, University of Cambridge, Downing Street, Cambridge CB2 3EJ, United Kingdom; 7 IUCN Global Species Programme, The David Attenborough Building, Pembroke Street, Cambridge CB2 3QZ, United Kingdom; 8 Nature-based Solutions Group, IUCN, 28 Rue Mauverney, 1196 Gland, Switzerland; 9 IUCN Species Survival Commission, Amphibian Specialist Group, Toronto M8W 1R2, Canada; 10 Royal Botanic Gardens, Kew, Richmond, Surrey TW9 3AB, United Kingdom; 11 Institute of Zoology, Zoological Society of London, Regent’s Park, London NW1 4RY, United Kingdom; 12 Department of Life Sciences, Natural History Museum, London SW7 5BD, United Kingdom; 13 IUCN Marine Biodiversity Unit, Global Species Programme/ Biological Sciences, Old Dominion University, Norfolk, Virginia, United States of America; 14 IUCN CI Biodiversity Assessment Unit, IUCN Global Species Programme, c/o Conservation International, 2011 Crystal Drive, Suite 500, Arlington, VA 22202, United States of America; 15 ARC Centre of Excellence for Environmental Decisions, Centre for Biodiversity and Conservation Science, The University of Queensland, 4072 Brisbane, Queensland, Australia; 16 School of Geography, Planning and Environmental Management, The University of Queensland, 4072 Brisbane, Queensland, Australia; 17 Luc Hoffmann Institute, WWF International, 1196 Gland, Switzerland; 18 Centre for Ecosystem Science, University of New South Wales, Sydney, New South Wales 2052, Australia; 19 School of Life Sciences, Arizona State University, P.O. Box 874501, Tempe, AZ 85287, United States of America; 20 IUCN Global Ecosystem Management Programme, The David Attenborough Building, Pembroke Street, Cambridge CB2 3QZ, United Kingdom; 21 Provita, Apdo. 47552, Caracas 1041-A, Venezuela; 22 Centro de Ecología, Instituto Venezolano de Investigaciones Científicas, Apdo. 20632, Caracas 1020-A, Venezuela; 23 Global Mammal Assessment Program, Department of Biology and Biotechnologies, Sapienza University of Rome, Viale dell'Università 32, I-00185 Rome, Italy; 24 Biodiversity Conservation Group, IUCN, 28 Rue Mauverney, 1196 Gland, Switzerland; 25 Critical Ecosystem Partnership Fund, 2011 Crystal Drive, Suite 500, Arlington, VA 22202, United States of America; 26 World Commission on Protected Areas IUCN, 64 Juniper Road, Chelsea, QC J9B1T3, Canada; 27 NatureServe, 4600 N. Fairfax Dr., Arlington, VA 22203, United States of America; 28 American Bird Conservancy, 1731 Connecticut Avenue, Washington DC 20009, United States of America; 29 New South Wales Office of Environment and Heritage, Hurstville, New South Wales 2220, Australia; Chinese Academy of Forestry, CHINA

## Abstract

Knowledge products comprise assessments of authoritative information supported by standards, governance, quality control, data, tools, and capacity building mechanisms. Considerable resources are dedicated to developing and maintaining knowledge products for biodiversity conservation, and they are widely used to inform policy and advise decision makers and practitioners. However, the financial cost of delivering this information is largely undocumented. We evaluated the costs and funding sources for developing and maintaining four global biodiversity and conservation knowledge products: The IUCN Red List of Threatened Species, the IUCN Red List of Ecosystems, Protected Planet, and the World Database of Key Biodiversity Areas. These are secondary data sets, built on primary data collected by extensive networks of expert contributors worldwide. We estimate that US$160 million (range: US$116–204 million), plus 293 person-years of volunteer time (range: 278–308 person-years) valued at US$ 14 million (range US$12–16 million), were invested in these four knowledge products between 1979 and 2013. More than half of this financing was provided through philanthropy, and nearly three-quarters was spent on personnel costs. The estimated annual cost of maintaining data and platforms for three of these knowledge products (excluding the IUCN Red List of Ecosystems for which annual costs were not possible to estimate for 2013) is US$6.5 million in total (range: US$6.2–6.7 million). We estimated that an additional US$114 million will be needed to reach pre-defined baselines of data coverage for all the four knowledge products, and that once achieved, annual maintenance costs will be approximately US$12 million. These costs are much lower than those to maintain many other, similarly important, global knowledge products. Ensuring that biodiversity and conservation knowledge products are sufficiently up to date, comprehensive and accurate is fundamental to inform decision-making for biodiversity conservation and sustainable development. Thus, the development and implementation of plans for sustainable long-term financing for them is critical.

## Introduction

We live in an era of unprecedented access to biodiversity and conservation data and information [1]. An enormous wealth of primary biodiversity data are collected around the world directly through field surveys or indirectly through remote sensing, and more recently through citizen science [2, 3]. To be useful, these primary data must be collated into databases, validated and processed into information that can be readily used to improve knowledge, and thus inform decision-making in policy and practice, as well as to transfer knowledge and enhance capacity building [4]. New technologies have facilitated the development of biodiversity information platforms that aim to make such information easily accessible to, and usable, by a wide range of audiences [5]. Shifting from potential usability by specialists to actual utility therefore requires the development of knowledge products. UNDP [6] describes good knowledge products as: relevant; based on an assessment of demand, audience needs, and unbiased evaluation; timely; clearly and consistently written and presented; developed through participatory processes; and easily accessible. In the context of biodiversity and its conservation, Brooks *et al*. [7] identify standards, governance, quality control, data, tools, and capacity building mechanisms as common components for four knowledge products: (1) The IUCN Red List of Threatened Species (which documents species extinction risk) developed by IUCN and a consortium of Red List Partners, including BirdLife International, Botanic Gardens Conservation International, Conservation International, Microsoft Research, NatureServe, Royal Botanic Gardens Kew, Sapienza University of Rome, Texas A&M, Wildscreen and the Zoological Society of London; (2) Protected Planet (incorporating the World Database on Protected Areas, which documents designation of areas for conservation) developed by IUCN and UNEP-WCMC; (3) the World Database of Key Biodiversity Areas (KBAs; which documents sites contributing significantly to the global persistence of biodiversity), developed by BirdLife International, the Critical Ecosystem Partnership Fund, IUCN and several other organisations that are currently joining together to establish a KBA Partnership; and (4) the IUCN Red List of Ecosystems (which documents risk of ecosystem collapse), developed by IUCN and a team of partners, including Provita, Centre for Ecosystem Science (UNSW Australia) and NSW Office of Environment and Heritage. These four knowledge products are in different phases of development that span from established and widely used in conservation practice and policy, to initial implementation (Table 1).

**Table 1:** Development status of the four knowledge products included in this study. A brief description of each knowledge product is available in [7].

*Knowledge product*	*Development of standards and processes*	*Development of data*, *tools*, *products and capacity*
The IUCN Red List of Threatened Species	[67, 68]	Advanced, datasets comprehensive globally for many taxa and countries
	Adopted by IUCN Council Decision C/51/35	
www.iucnredlist.org		
Protected Planet	[69, 70]	Advanced, datasets comprehensive globally for most countries.
www.protectedplanet.nethttp://www.protectedplanet.net/	United Nations Economic and Social Council (ECOSOC) Resolution 713 (XXVII)	
	Endorsed by IUCN Resolution WCC-2012-Res-040	
The World Database of Key Biodiversity Areas	[53, 71]	Datasets for some components of biodiversity (i.e. IBAs, AZE sites); other components in progress. Database, tools and capacity development for KBA identification are being improved and expanded.
	Adopted by IUCN Council Decision C/88/25.	
www.birdlife.org/datazone/site		
www.zeroextinction.org/search.cfm		
IUCN Red List of Ecosystems	[57, 61, 72]	Some countries and regions completed. Pilot databases developed, tools and capacity building support are available; additional resources are underway.
	Adopted by IUCN Council Decision C/83/17.	
www.iucnredlistofecosystems.org		

These four knowledge products are widely applicable in conservation decision-making in at least four contexts. First, they are fundamental for tracking progress towards international biodiversity commitments such as the Millennium Development Goals [8, 9, 10], the Aichi Biodiversity Targets of the 2020 Strategic Plan for Biodiversity [11, 12, 13], and the emerging Sustainable Development Goals [14, 15, 7]. Notably, around a third of the indicators used to assess current status of Aichi Biodiversity Targets draw information from The IUCN Red List of Threatened Species, Protected Planet, and/or the World Database of Key Biodiversity Areas [16, 13]. Second, they are increasingly used by the private and banking sectors to assess potential biodiversity risks associated with their activities [17, 18]. Third, they are extensively used to inform conservation strategies and plans, guiding, for example, investments by the Global Environment Facility and the Critical Ecosystem Partnership Fund [19, 20, 21], as well as numerous National Biodiversity Strategies and Action Plans under the Convention on Biological Diversity [22] and global and regional environmental assessments [23]. Fourth, and possibly most importantly, they are used to inform local land/seascape planning decisions around the world, although this is often hard to document (e.g., [24], [25], [26], [27]). While not all four knowledge products may have been used yet in all four contexts they are certainly designed to do so.

However, the delivery of all four of these knowledge products faces two major challenges. First, none have reached their stated goals for comprehensive data coverage to build a robust baseline. These shortfalls in coverage leave serious biases in the datasets; e.g., in geographic and taxonomic coverage for The IUCN Red List of Threatened Species and for the World Database of Key Biodiversity Areas; in taxonomic and geographic coverage for the IUCN Red List of Ecosystems; and in governance types of protected areas documented by Protected Planet. Second, their utility is dependent on their quality and currency [28]. This means they require continuous maintenance and updating to reflect, for example, the impacts of changing taxonomy or protected area legislation, emerging threats or status changes resulting from conservation and development action, and to keep abreast of innovations in technology.

The overall cost of such development and maintenance of these knowledge products has not been assessed comprehensively. The few studies available have focused on The IUCN Red List of Threatened Species (e.g. [29], [30], [28], [31], [32], [33]). Most of these studies typically included only the cost of conducting species extinction risk assessments, but not the costs of maintaining structures and processes associated with the delivery and publication of such assessments. There have also been a number of studies quantifying the cost of conservation actions (e.g.,[34]) and the cost of achieving conservation targets (e.g., [35], [36]), but, again, these do not include estimates of the cost of knowledge generation from primary data necessary to guide the planning and monitoring of such actions and achievement of targets.

The financial cost of developing and maintaining these biodiversity and conservation knowledge products is not trivial. Quantifying this cost is fundamental for those who invest in their delivery, to ensure sustainable financing in the long term, and to allow exploration of ways to minimise these costs by improving the efficiency of data collection, validation processes, and dissemination, through, for instance, integration in unified technological platforms [28]. Given the widespread use of these knowledge products to inform policy and decision making across sectors and at global, national and regional scales, it is equally important to understand their value as a global good, and to put in perspective the relative benefit of the conservation outcomes with respect to the investment in developing and maintaining knowledge products.

There is a clear a gap in the understanding of the actual costs associated with the delivery of global biodiversity datasets, their funding sources, and the relative importance of these investments. To fill this gap, we assess the costs of developing and maintaining The IUCN Red List of Threatened Species, Protected Planet, The World Database of Key Biodiversity Areas, and the IUCN Red List of Ecosystems. First, we evaluate the funding invested until 2013 and the annual costs in 2013 for each knowledge product (excluding the IUCN Red List of Ecosystems for which annual costs were not possible to estimate for 2013), along with the sources of this financing (by sector) and types of expenditure. We explain the scope of this study and we describe how the data on costs were collected and standardised. Second, we estimate the funding needed to reach pre-defined baselines by 2020 for each of these knowledge products, and the estimated annual costs of maintaining such expanded data coverage. Finally, we discuss the results in the context of ensuring long-term sustainability and delivery of biodiversity and conservation knowledge products to inform decision-making in diferent contexts, and show that the funding needed to do this is relatively low compared with other similar processes.

## Methods

### Scope of the study

**Fig 1:**
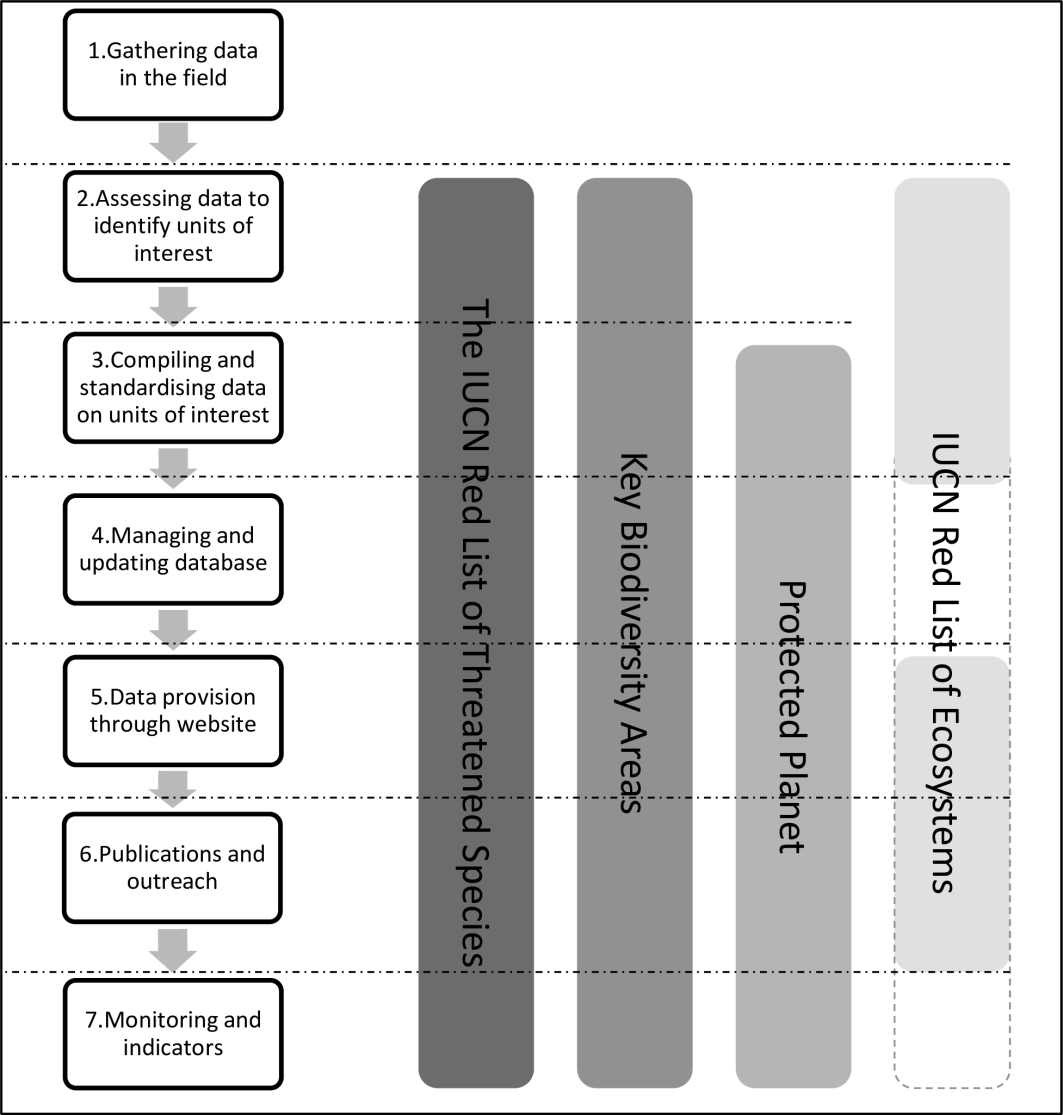
Different stages of knowledge generation and dissemination covered by the four knowledge products for which costs are included in this review. The coloured bars show which stages are covered by each knowledge product currently which were costed in the study. In 2013 only IBAs, AZE and KBAs identified through CEPF hotspot ecosystem profiling processes cover stages 4, 5 and 7.The IUCN Red List of Ecosystems will cover stages 4 and 7 in the near future; an extensive list of technical resources is available at www.iucnredlistofecosystems.org but no spatial data or indicators are yet available.

Biodiversity knowledge products are compiled and maintained in diverse ways, with multiple phases of data collection and processing, each associated with a cost (Fig 1). Typically, primary biodiversity data, which underpin the knowledge products, go through several stages of data collation, compilation, validation, and quality control before knowledge products are made available to the wider community and general public in various forms (databases, spatial data, assessments, indicators, web services, publications, etc.).

Here, we focus on the costs of the secondary processes that are directly and completely devoted to the delivery of the knowledge products (see Table 2 and S3 Table). We do not include the costs of collection of primary data [37] which, while essential for the delivery of the knowledge product, precede work on the knowledge products themselves and are not exclusive to them. The most obvious example of primary cost is gathering data in the field. For example, we did not include the costs of establishing, mapping, digitizing, and maintaining national databases of protected areas or the cost of surveying, classifying and mapping ecosystems for the Red List of Ecosystems; rather we included the costs of aggregating these data centrally through workshops and direct contact with governmental protected area agencies, and subsequent quality checking and revision before making them available online. Similarly, for The IUCN Red List of Threatened Species, we did not include the costs of establishing, digitizing and maintaining specimen locality databases. We also excluded the costs of maintaining networks supporting data collection such as the IUCN Species Survival Commission (IUCN SSC) network of Specialist Groups and Red List Authorities which support The IUCN Red List of Threatened Species; the network of BirdLife Partners worldwide who lead identification and monitoring of Important Bird and Biodiversity Areas (the subset of KBAs identified using data for birds [26]; or the IUCN World Commission on Protected Areas (IUCN WCPA) network supporting the development of the WDPA.

**Table 2:** Categories, subcategories and funding sources classification used to categorise costs.

*Cost Category*	*Definition*
Concept development	Cost of the process of developing the knowledge product concept (e.g. development of IUCN Red List of Ecosystems Categories and Criteria)
Data development and assessments	Cost of compilation of primary data and assessment using the knowledge product standards (e.g. first global bird extinction risk assessment)
Maintenance of standards and systems	On-going costs per year of managing and maintaining data standards and information systems such as databases and websites (e.g. maintenance of The IUCN Red List of Threatened Species database).
Monitoring and indicator development	On-going costs per year of repeat assessments to maintain coverage of data compiled, to derive indicators (e.g. Global Protected Area Coverage).
*Cost Sub-category*	*Definition*
Personnel time	Remunerated time of individuals
Travel and workshops	Travel and accommodation expenses
Infrastructure	Maintenance of offices, information systems information systems such as databases and websites.
Publications and outreach	Reports, books, leaflets. It does not include cost of producing scientific papers.
*Funding Source*	*Examples*
Governments	Ministry for the Environment, Germany
Financial Institutions	World Bank
Intergovernmental organizations (IGOs)	United Nations Environment Programme
Multilateral donor	Global Environment Facility (GEF)
Non-Governmental Organizations (NGOs)	Birdlife International, Conservation International, academia
Philanthropy	MacArthur Foundation, MAVA Foundation, Gordon and Betty Moore Foundation
Private Sector	Shell, Rio Tinto
Voluntary time	Non-remunerated time spent in reviewing assessments, providing and validating data, attending meetings and workshops, etc.

These processes are important for numerous reasons and applications above and beyond the delivery of biodiversity and conservation knowledge products, and so including their costs here would inflate our estimates inappropriately. However, it must be emphasized that these processes, worth many millions of dollars per year (see, e.g., [38], [39]) and are critical to proper establishment, development and ongoing maintenance of knowledge products. Therefore, the costs reported herein for knowledge product delivery represent a fraction of the total cost to populate knowledge products with data (e.g. Fig 1, stage 1). Similarly, we did not assess the costs or benefits that result from use of knowledge products which are typically incurred by end-users of the information (e.g., conservation actions such as protected area establishment and management, interventions for threatened species, changes to land use policies, etc.).

Finally, we excluded the costs of national assessments which follow national standards, such as national red lists of species or designating and delineating new protected areas. Some national species assessments, for example, do contribute to the global IUCN Red Lists of Threatened Species and Ecosystems when a nationally endemic species or ecosystem type is assessed using the IUCN Categories and Criteria and the data are shared with IUCN [40, 27], but the processes and costs of generating a national red list usually occur and are borne independently of the global IUCN Red Lists [25, 41].

### Compiling costs

Data collection for this review was undertaken from August 2013 to November 2014. We obtained and collated costs of: 1) developing and maintaining knowledge products from the earliest year for which data on costs were available (for each product) up until December 2013; 2) annual costs of maintaining data, structures, and processes in 2013; and 3) projected future expenditure to achieve pre-defined baselines by 2020.

To compile the past, present and future costs in a standardised way we classified costs by knowledge product stage, cost type, and funding source (Table 2). We contacted individuals who manage or have managed knowledge product development processes (henceforth referred to as *study data providers*), and asked them to provide the relevant cost data. A spreadsheet with options and specific instructions were created for collection of data in a standardised way, to facilitate subsequent analyses. The spreadsheet included drop-down lists and free-text fields which allowed study data providers to classify each cost under each of the pre-defined expenditure categories and subcategories, and funding sources. Study data providers were requested to provide minimum and maximum estimates where precise values were not known, currency and year of currency, and minimum and maximum volunteer time invested. All files received were then merged into a single database which included all costs collected for each of the four knowledge products. Finally, to account for inflation and standardise estimates, all costs were converted to 2014 US$ (see S1 Table).

### Addressing data gaps

We anticipated that we would be unable to obtain complete data on all of the past costs for each knowledge product (particularly for processes and components dating from further in the past). While the origins of The IUCN Red List of Threatened Species date back to the early 1960s [42], very little information was available on the costs of development of The IUCN Red List of Threatened Species methodology and infrastructure prior to 1999, except for the costs of assessing all birds globally, which date from 1985. Similarly, we compiled the costs associated with the establishment of Protected Planet since 1981, when the first digital version of the World Database on Protected Areas (WDPA)–the database underpinning Protected Planet since 2008 –was created. However, the history of the WDPA goes back to 1959, when the United Nations (UN) Economic and Social Council called for a list of national parks and equivalent reserves in their natural state (Resolution 713 (XXVIII)), which was then developed with considerable support from the IUCN WCPA network. Therefore, the financial and voluntary contributions from IUCN WCPA to the development and compilation of protected areas information before the establishment of a digital database on protected areas were not accounted for.

To estimate the total funds invested in knowledge products until 2013, we compared the number of units of assessment for which we obtained cost estimates (e.g., species in The IUCN Red List of Threatened Species with a known assessment cost) with the total number of assessment units in the database (e.g. total number of species on The IUCN Red List of Threatened Species) in December 2013 and scaled the total cost estimate proportionately (see Table 3). This assumes that the volume of available data for compilation, standards, methods and effort involved in assessments did not change through time. This is not the case especially for species assessments where the methods applied were different as the system used for assessing risk for extinction of species was updated in 1994. It is therefore likely that this have led to some under-estimation of earlier costs due to changes in criteria and increasing rigour in standards of assessment and documentation.

**Table 3:** Summary of data collection for all four knowledge products. The table summarises which costs were collected for each of the four knowledge products and how much of the total number of assesments, available in December 2013, these represent. In cases where 100% of the costs were not collected, the total sum for each knowledge product was increased propotionally to reach 100%.

*Knowledge product*	*Organizations that provided data for this study*	*Units*	*Number of units*	*Period covered*	*Percentage of dataset (December 2013)*
The IUCN Red List of Threatened Species^1^	IUCN, BirdLife International, University of Rome, Zoological Society of London, NatureServe, Royal Botanical Gardens Kew, and Natural History Museum London.	Species assessments and associated spatial and tabular data	**76,068**	1985–2013	**68%**
Protected Planet^2^	UNEP-World Conservation Monitoring Centre and IUCN	Protected areas spatial and tabular data	**214,000**	1981–2013	**100%**
The World Database of Key Biodiversity Areas (KBAs)^3^	BirdLife International, Alliance for Zero Extinction, IUCN, and CEPF.	Key Biodiversity Areas spatial and tabular data	**17,732**	1979–2013	**88%**
IUCN Red List of Ecosystems^4^	IUCN, Provita, Instituto Venezolano de Investigaciones Cientificas (IVIC), and the Centre for Ecosystem Science, University of New South Wales, Australia	Ecosystem assessments and associated spatial and tabular data	**616**	2004–2013	**100%**

^***1***^The dataset assessed was all species published on www.iucnredlist.org by the end of 2013 including re-assessments.

^2^The dataset assessed was the December 2013 version of World Database on Protected Areas.

^***3***^The dataset assessed was the World Birds and Biodiversity Database.

^4^The dataset assessed was all ecosystem assessments completed or about to be completed by end of 2013.

Our data comprised 875 cost records for all four knowledge products sourced from 11 study data providers (Table 3). As anticipated, this does not represent 100% of the costs for each knowledge product (Table 3). However, we covered the costs for conducting 76,068 species assessments (relating to 39,533 species, some of them assessed multiple times) between 1985 and 2013. This represented 68% of species assessments on The IUCN Red List of Threatened Species as of December 2013, hence we proportionately rescaled the cost to match 100% of the species. Similarly, our data for KBAs from 1979–2013 accounted for 88% of existing KBAs, and we rescaled the cost to represent 100% of exisiting sites. Aside from the important exclusions outlined in these Methods–notably the exclusion of primary data generation and compilation costs by national agencies–all of the secondary known costs associated with producing Protected Planet and the global IUCN Red List of Ecosystems were collected, so these were not increased other than by adjusting all the costs to account for inflation.

### Accounting for voluntary time

Voluntary time (including time donated to knowledge product delivery that is paid for by other institutions, universities, museums, governments, departments and NGOs) plays a crucial role in the production of biodiversity information. For example, more than ten thousand individuals through the IUCN Commissions and many more outside the Commissions, have provided voluntary input to the delivery of knowledge products [7]. These range from individuals compiling information to experts participating in workshops, reviewing assessments, providing data, and contributing to technical committees or governing bodies. Here, we recorded volunteer time invested in each knowledge product in terms of the number of working days (one working day equalling eight hours) and considering 240 working days per year (52 weeks per year minus four weeks of holidays at five working days per week).

In many instances (if not most), because it was not an official requirement in project management, volunteer time invested had not even been accounted for or was not consistently recorded, making it difficult to apply a valid method. For these reasons we are likely to have underestimated substantially the total extent of volunteer time invested. We acknowledge that our estimates are uncertain, do not necessarily reflect the variation in any given process, and in some instances they may only capture a small subset of the input invested into assessments. For example, for amphibian assessments on the IUCN Red List considered in this paper, the estimate of volunteer time is solely based on workshop participation from 2001–2004 (see S2 Table and S3 Table); however, as mentioned above, this is just one form of input volunteers contribute to assessments, and it does not capture the full range of work required for these processes.

Acknowledging the complexity of measuring the value of volunteer time, Salamon *et al*. [43] recommend the use of the replacement cost method which estimates the value of the work that the volunteer performs based on market wages. The enormous variation in expertise, experience, and geographic location of individuals providing it makes assiging a monetary value to the time invested by volunteers in the development and maintanance of biodiversity knowledge products highly complex. Nevertheless, we assigned a value to volunteer time using a higher and lower estimate attempting to assign a financial value to the known volunteer contributions based on different levels of experience and expertise. The higher estimate was US$30.97 per hour, which is the mean hourly rate of a conservation scientist according to the U.S. Bureau of Labor Statistics [44]. The lower estimate is US$23.07 per hour, a generic volunteer time estimate provided by Independent Sector [45]. Two levels of uncertainty are incorporated through this approach. First, the uncertainty reflected in the maximun and minimum working days assigned in each estimate. Second, uncertainty on the monetary value assigned to those estimates.

### Reaching pre-defined baselines by 2020

For each knowledge product we estimated: 1) annual maintenance costs for each year until 2020, considering inflation and annual incremental costs; 2) additional one-off costs required to reach stated target ‘baselines’ by 2020 (e.g., additional investment to improve infrastructure, new assessments to be undertaken, updating old assessments). We did this by drawing from information compiled from past and annual costs of knowledge products, plus additional future projections documented by the study data providers, specifically:

- **The IUCN Red List of Threatened Species:** we followed Stuart *et al*. [30] who propose the assessment of 160,000 species based on a *“subset of species broadly representative of biodiversity as a whole*”, including sampled assessments for selected groups [46], to enable better conservation and policy decisions at a global level.
- **Protected Planet:** We assessed the costs of implementing the Protected Planet strategy by 2020. This includes re-structuring the Protected Planet website to improve data dissemination, expanding the scope of the WDPA to record information on “other effective area-based conservation measures” [47], integrating the Global Database on Protected Area Management Effectiveness [48], and enhancing interoperability with other relevant datasets such as the other knowledge products discussed here.
- **The World Database of Key Biodiversity Areas:** We assessed the costs of expanding the KBA dataset, documenting existing KBAs for other taxonomic groups and criteria, and the predicted costs of a governance structure to manage the KBA program in the future. Two global networks of KBAs have been systematically identified to date: c. 12,800 Important Bird and Biodiversity Areas derived from bird data (IBAs; [49], [26]) and 587 Alliance for Zero Extinction sites derived from data for mammals, birds, a small sample of selected reptiles, conifers, reef-building corals, and amphibians (AZEs; [50]). In regions where KBAs have been identified for multiple taxonomic groups [51], IBAs comprised 68% of all KBAs identified [26]. We used this percentage to extrapolate from the total number of recognised IBAs to the potential total number of KBAs that may be identified globally, although we recognise that IBA density varies in different regions of the world, e.g. higher in Europe and lower in Australia [52]. We then assessed the costs of identifying these new sites, the costs of documenting all existing sites for a wider range of taxa and under new criteria in the forthcoming KBA standard [53] drawing from the known costs of similar processes [54]. This approach is likely to result in an underestimate because IBAs are likely to contribute a smaller proportion of all KBAs, especially in freshwater [55] and marine ecosystems [56]. Moreover, the identification of KBAs for multiple taxonomic groups (see [51] and references therein), although based on the best available data at the time, has only covered a subsample of plant and invertebrate groups.
- **IUCN Red List of Ecosystems:** Although there is a stated aim to complete the global IUCN Red List of Ecosystems by 2025 [57], including assessment of all terrestrial, freshwater, marine and subterranean ecosystems worldwide, for the purposes of alignment and comparability in our study, we regarded completion of the first global IUCN Red List of Ecosystems by 2020 as a pre-defined baseline. Indeed, if all the resources and capacity required (as estimated in our study) were to be made available in good time, the original 2025 target [57] could be achieved earlier. We estimated the costs for reaching this endpoint based on the costs of managing and upscaling assessment processes that are underway and the predicted cost of establishing and managing an online database for a global IUCN Red List of Ecosystems and putting in place governance structures and training programmes. Further information about how the projections were calculated is available in S2 Table.

## Results

### Costs up to and including 2013

In total, at least US$160 million (range: US$116—US$203 million) and at least 293 person-years of volunteer time (range: 278–308 year), valued at US$14 million (range: US$12 million–US$16 million), have been invested in the four knowledge products between 1979 and 2013 (Table 4). Total costs have been greatest in KBAs (over half of the total investment in the knowledge products), followed by The IUCN Red List of Threatened Species (22%), Protected Planet (12%), and IUCN Red List of Ecosystems (4%).

**Table 4:** Overall funds (midpoints) and volunteer days invested in the four knowledge products between 1979 and 2013, and annual cost in 2013. The mid-point is the equidistant point between the maximum and minimum values. Full details are available in S4 Table.

*Knowledge product*	*One-off costs*		*Annual cost in 2013*	
	*Total funds*	*Volunteer time*	*Total funds*	*Volunteer time*
The IUCN Red List of Threatened Species	US$34,935,095	209 person-years US$8,788,075	US$4,785,729	2,474 days US$504,085
Protected Planet	US$19,055,847 US$	5 person-years US$242,492	US$861,000	35 days US$7,132
The World Database of Key Biodiversity Areas	US$99,106,414	78 person-years US$3,269,511	US$856,414	454 days US$92,492
IUCN Red List of Ecosystems	US$6,706,400	2 person-years US$80,899	Not available	Not available
Total	**US$159,803,756**	**293 person-years US$14,335,785**	**US$6,503,144**	**2,963 days US$603,709**

**Fig 2:**
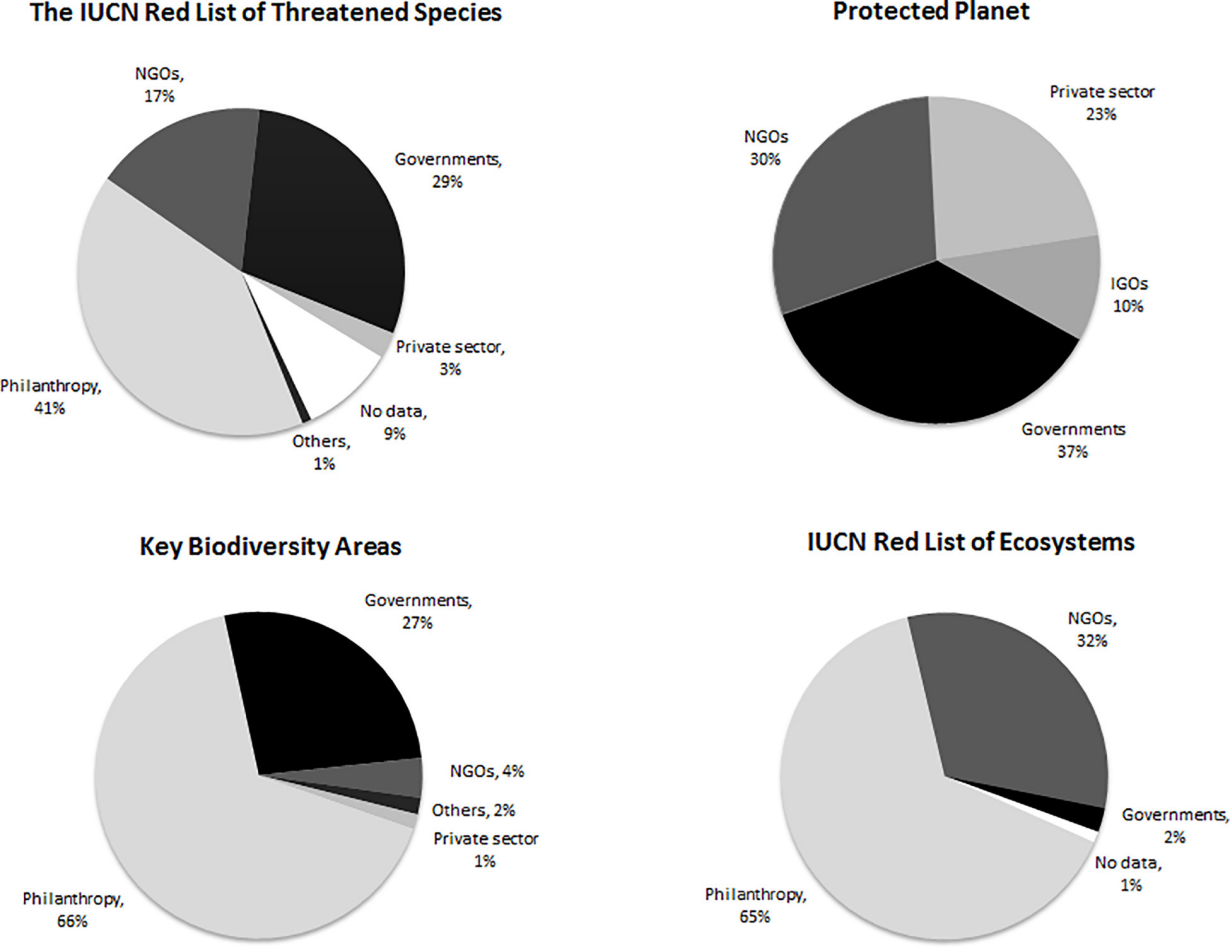
Sources of funding (midpoints of estimates) until 2013 for all knowledge products. Others include multilateral donors and financial institutions.

More than half of the funding (53%) took the form of philanthropic donations from foundations and individuals (Fig 2). Intergovernmental Organizations, Non-Governmental Organizations and Governments have together contributed a combined total of 43% to the development and maintenance of knowledge products. The private sector, multilateral donors and financial institutions have contributed with just5% of the costs of development and maintenance of knowledge products.

**Fig 3:**
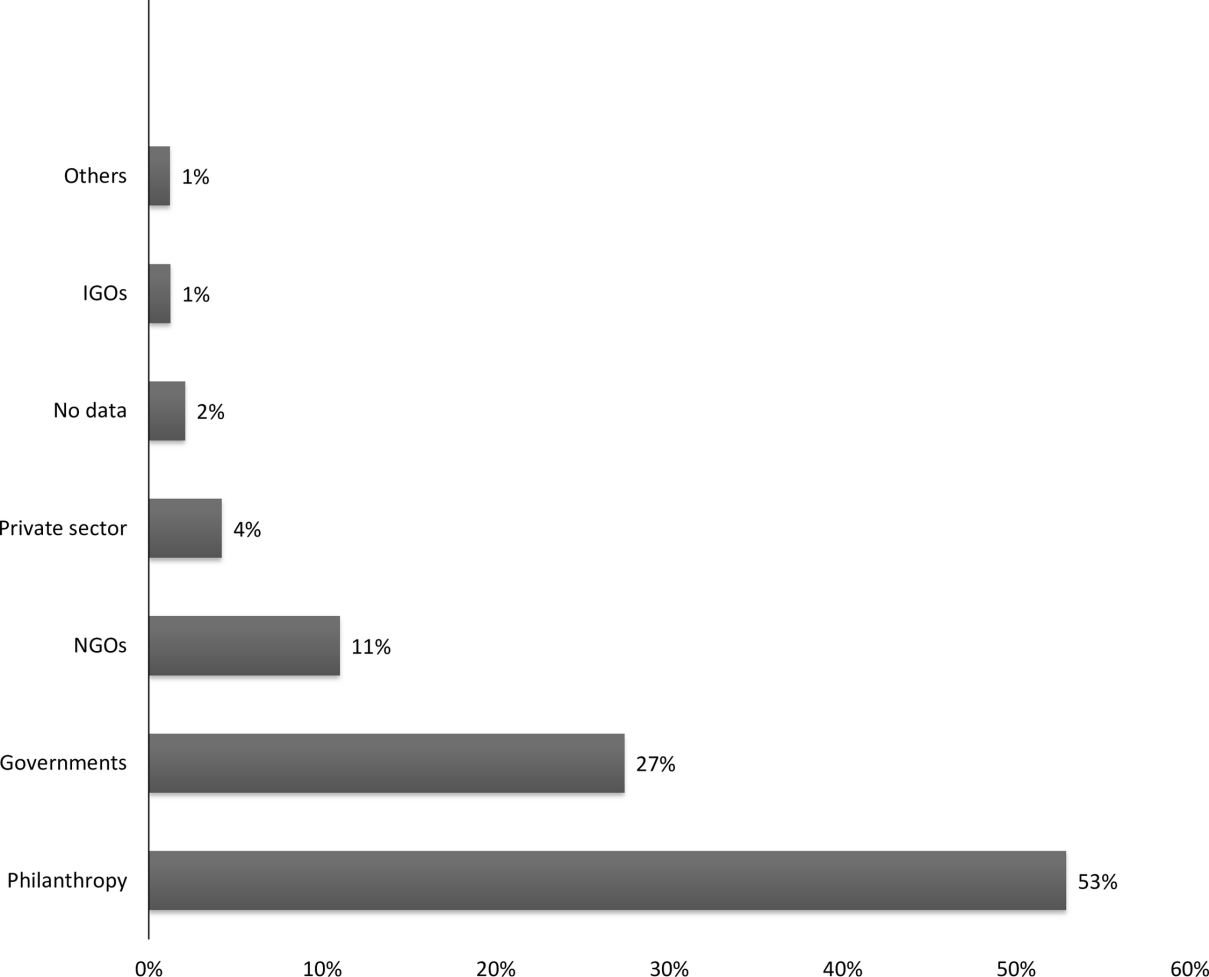
Sources of funding (midpoints of estimates) invested until 2013 for each knowledge product.

The relative importance of different sources of funding varies between the individual knowledge products (Fig 3). The IUCN Red List of Threatened Species, IUCN Red List of Ecosystems, and The World Database of Key Biodiversity Areas have each received more support from philanthropy than from any other single source, with governments representing an important secondary source of finance for both The IUCN Red List of Threatened Species and The World Database of Key Biodiversity Areas, while NGOs play a similar role with respect to the IUCN Red List of Ecosystems. Protected Planet has a strikingly different funding profile, being primarily funded by NGOs and Government sources, with the private sector as an important secondary funding source. This funding profile is a result of the history of Protected Planet, reflecting diverse intial number of sources supporting this knowledge product in the first decades of development, in partnership with IUCN WCPA and the subsequent contributions of the Proteus Partnership (www.proteuspartners.org) in maintaining the system since 2006.

**Fig 4:**
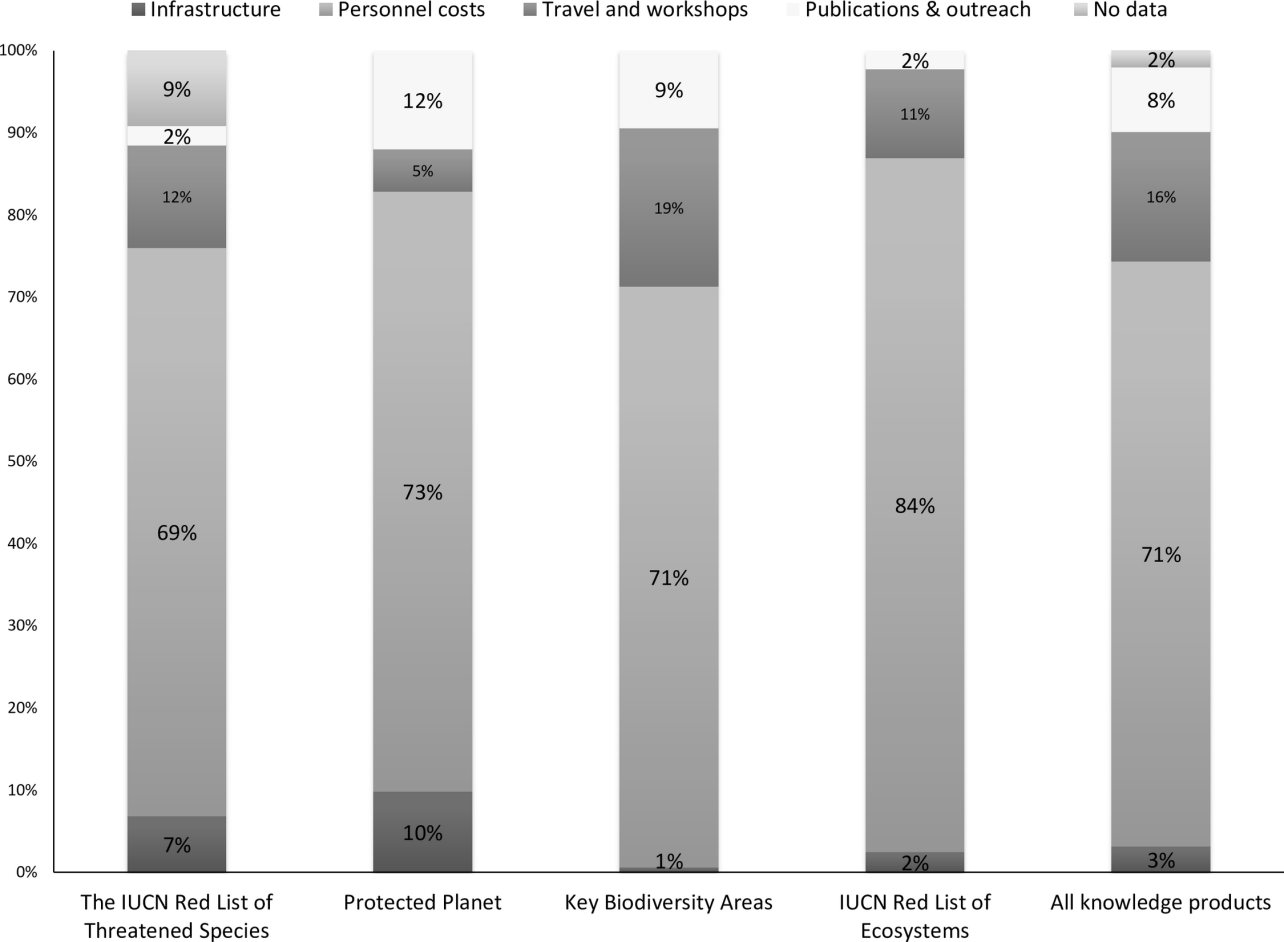
Categories of costs (midpoints of estimates) for funding invested until 2013 for each knowledge product.

Personnel time is the major expense for all four knowledge products, accounting for 71% of overall costs (Fig 4). The importance of other cost sub-categories varies and depends on the processes behind each knowledge product. For example, more funds have been committed to infrastructure for knowledge products that have fully developed online platforms to access and download data, such as The IUCN Red List of Threatened Species (7% of overall costs) and Protected Planet (10%). Such systems are less well developed for The World Database of Key Biodiversity Areas (1%) and IUCN Red List of Ecosystems (2%) so far, due to their earlier stage of application.

### Annual costs in 2013

The delivery of knowledge products involves a series of common processes (Fig 1), but the scale of these efforts and the way the processes are managed are very different between knowledge products. Accordingly, annual costs in 2013 differed widely for the different knowledge products: the estimated annual cost in 2013 was US$4.7 million for The IUCN Red List of Threatened Species, US$861,000 for Protected Planet, and US$856,000 for Key Biodiversity Areas (Table 4). This amounts to a total of US$6.5 million per year. In 2013, the global database for the IUCN Red List of Ecosystems was still under development, and therefore annual costs of maintaining data, structures and processes in 2013 could not be accurately estimated.

### Reaching pre-defined baslines by 2020

**Fig 5:**
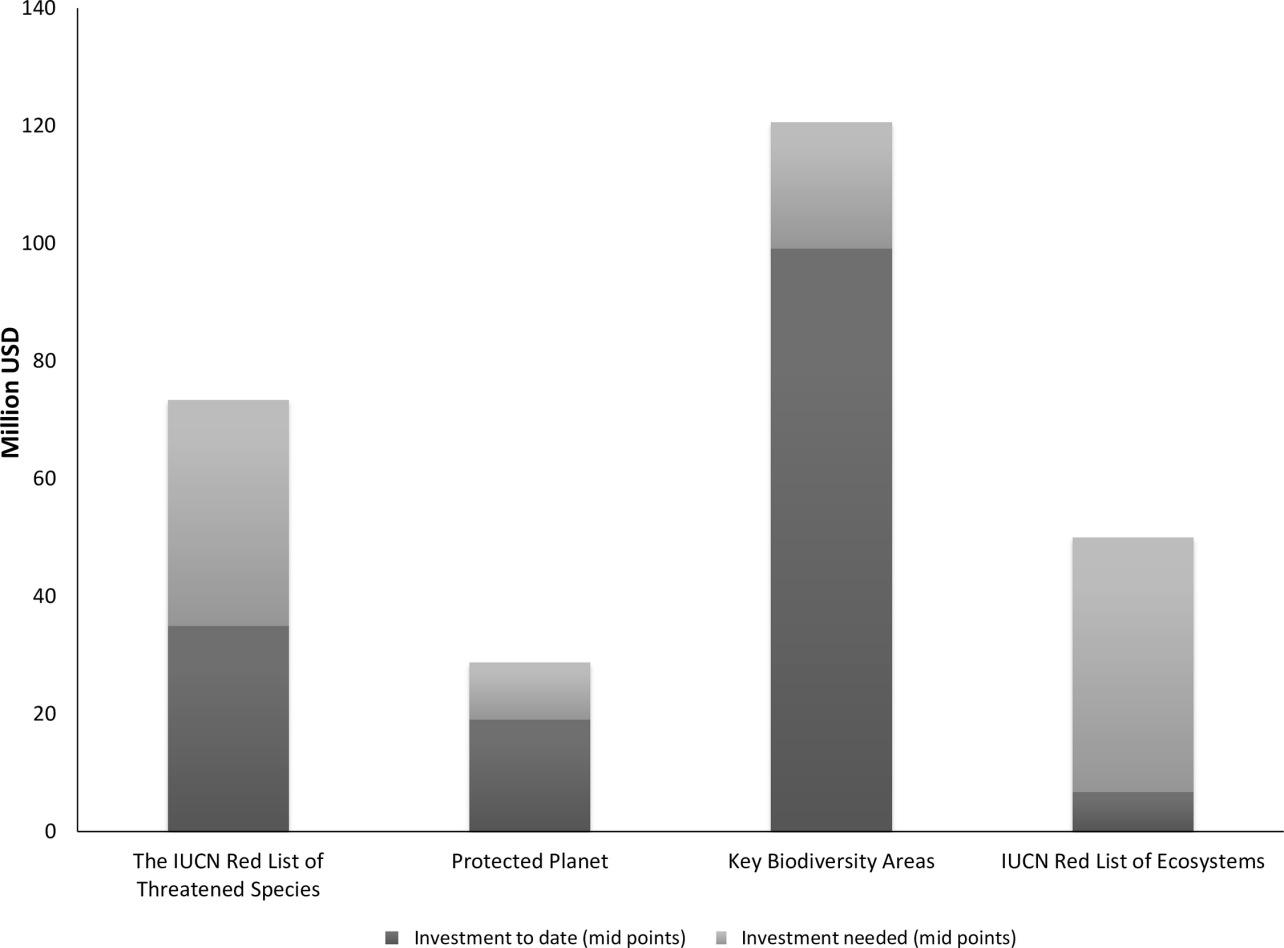
Estimated costs to reach pre-defined baselines by 2020 for each knowledge product.

Overall, an additional US$103 million in one-off investments would be needed to reach pre-defined baselines for the four knowledge products by 2020 (Fig 5). However, this amount is not distributed evenly between knowledge products because they are in different stages of development. For instance, an additional US$38 million will be needed to deliver the additional c. 85,000 species assessments on The IUCN Red List of Threatened Species necessary to provide a “Barometer of Life” [30] and maintain the database structures and processes to manage these by 2020. Implementing the Protected Planet strategy will require around US$10 million, while the cost for reaching a baseline for The World Database of Key Biodiversity Areas is estimated at US$21million. Finally, US$43 million was estimated to be required to complete a global IUCN Red List of Ecosystems by 2020. Once these baselines are achieved by 2020, the annual investment required thereafter to maintain them will be US$5.4 million for The IUCN Red List of Threatened Species, US$1.9 million for Protected Planet, US$2 million for The World Database of Key Biodiversity Areas and US$3.7 million for the IUCN Red List of Ecosystems.

## Discussion

To fill in an identified research gap, here we assess the cost of developing and maintaining four global biodiversity datasets: The IUCN Red List of Threatened Species, Protected Planet, The World Database of Key Biodiversity Areas, and the IUCN Red List of Ecosystems. We estimate that US$160 million (range: US$116–204 million), plus 293 person-years of volunteer time (range: 278–308 person-years) valued at US$ 14 million (range US$12–16 million), were invested in these four knowledge products between 1979 and 2013. In addition, US$6.5 million in total (range: US$6.2–6.7 million) were needed annually to maintain data and platforms for The IUCN Red List of Threatened Species, Protected Planet, and The World Database of Key Biodiversity Areas. Finally, we estimated that an additional US$114 million will be needed to reach pre-defined baselines of data coverage for all the four knowledge products, and that once achieved, annual maintenance costs will be approximately US$12 million.

### Differences between biodiversity knowledge products

Our results provide unique insight into the costs associated with the development and maintenance of some of the most extensively used biodiversity knowledge products ([58, 7] and S5 Table). The relative differences in total funds invested until 2013 and annual costs in 2013 between the four knowledge products are explained by the differences in the processes by which they are derived, as well as their differing stages of development.

#### The IUCN Red List of Threatened Species

The costs until 2013 and annual costs in 2013 for this knowledge product include both new assessments and re-assessments (to be carried out every 10 years) (Fig 1; stage 2). Our finding that US$38 million of investment will be required to attain the 160,000 species coverage for the “Barometer of Life” is broadly consistent with Stuart et al.’s [30] estimate of US$60m, given that 20,000 previously-unassessed species have been added to The IUCN Red List of Threatened Species over the last five years.

#### Protected Planet

The investments until 2013 and annual investment in 2013 in Protected Planet reflect global compilation, data management, and dissemination costs alone (Fig 1; stages 3–7), explaining their relatively modest costs. Now that the documentation, including boundary polygons, of protected areas managed by governments has plateaued over time [12], the bulk of the effort facing the delivery of Protected Planet will be to establish mechanisms to better document protected areas governed by non-governmental sectors, such as the various types of Indigenous and Community Conserved Areas (ICCAs) and privately protected areas, to track degazettal, downlisting and downsizing, as well as the inclusion of “other effective area-based conservation measures”, and improving user experience and access to more comprehensive information through Protected Planet. The establishment, management, and associated documentation of protected areas is a national process, and, although extremely important because of their role in generating primary data for Protected Planet, these national costs have been evaluated elsewhere (e.g., [34], [59], [35]) and as explained in the Methods are beyond the scope of this study (Fig 1; stages 1 and 2).

#### The World Database of Key Biodiversity Areas

That the KBA knowledge product had received the greatest investment until 2013 reflects the fact that this includes the costs of mainly IBA identification within each of approximately 200 countries and territories, as well as global coordination and compilation. It is also important to note that the KBA knowledge product is, to a great extent, dependent on the other three knowledge products considered here for its application (with both Red Lists necessary to allow identification of sites holding significant levels of Threatened biodiversity, and Protected Planet supporting site delineation for protected KBAs). Once KBAs have been identified, the cost of ongoing re-assessment is much lower than of initial identification and delineation (but note that this does not include the essential costs of field data collection for ongoing monitoring; [60]). The estimated US$21m cost to identify and document a comprehensive network of KBAs by 2020 is an underestimate, given the as-yet unknown extent to which limited KBAs identified so far in freshwater and marine environments are reflective of those for all biodiversity, and the fact that many sites are still to be documented for other largely terrestrial taxonomic groups.

#### The IUCN Red List of Ecosystems

This is a new knowledge product [61, 27], so its costs until 2013 have been small and have not yet fully covered components such as database maintenance, online dissemination, or indicator development (Fig 1; stages 4, 5, and 7), and it is not yet meaningful to derive an annual cost. This also explains the relatively large investment which will be required to deliver a baseline for achieving a global IUCN Red List of Ecosystems by 2020.

### Diversifying funding sources for knowledge products

Perhaps our most surprising result in considering investments until 2013 is the lack of diversification in funding sources. While the combined contributions of NGOs, intergovernmental organisations, and governments account for 43% of funding for knowledge products in the last four decades, more than half of the funding has come from philanthropic sources. This dominance reflects both a high reliance on project funding and the often catalytic role of philanthropic funding. However, the distribution of funding sources seems heavily skewed given the extensive use and uptake of knowledge products to inform practice and policy across sectors, and users of knowledge products seem to contribute very little to their financial sustainability. In particular, contributions from the private sector could be greatly increased, given the currrent importance of three knowledge products (all except the Red List of Ecosystems which has not yet been incorporated in IFC’s standards) in maintaining the International Finance Corporation’s Performance Standard 6, which is used in turn by all the Equator Principles Banks, and, in aggregate, guides tens of billions of dollars of annual private sector loans [17]. The establishment of the Proteus Partnership, and of the Integrated Biodiversity Assessment Tool (www.ibatforbusiness.org), which makes the knowledge products available for commercial use through data licensing, represents an initial step in this direction for support. Proportionate contributions from governments should also be increased [34], considering the importance of the knowledge products in supporting national-level planning as well as tracking progress towards countries’ international commitments and contributing to knowledge transfer [4].

By contrast, the allocation of investment until 2013 across expenditure categories is rather similar among the four knowledge products, primarily driven by personnel costs, accounting for nearly three-quarters of all expenses. This reliance on human capital is further underscored by the enormous volunteer contributions into the process, notwithstanding its underestimation here (293 person-years–US$14 million). Much of this volunteer effort comes from experts belonging to IUCN’s Species Survival Commission working on species assessments or experts from the BirdLife International partnership working on IBA identification and documentation. Relatively greater investments in technology might increase efficiency of the process (e.g., increasing the role of online consultation rather than workshops for reassessing species’ extinction risk; [28]) but we suspect that personnel costs (and volunteer contributions) are a fundamental reflection of the human capacity needed for biodiversity and conservation assessments, and the data collection and management underlining them to ensure they are robust.

### Making knowledge products more efficient

The database generated by this project incorporates valuable information to assess more in-depth aspects of the delivery of biodiversity and conservation knowledge products, such as comparing the efficiency of different processes within each knowledge product. For example, the cost of assessing species varied greatly between taxonomic groups (making it difficult to arrive at a single dollars per species assessment cost), which in part reflects different approaches applied to collate data and apply them to the IUCN Red List Categories and Criteria and, which have different costs [28] and the extent to which training and capacity-building actions are incorporated into the assessment process. Our data could also offer insights on current costs which could be used for the development of innovative approaches to, for example, reduce costs of data compilation and validation, allowing greater investment in interpretation, analyses and tool development. Another issue which these data would help to inform concerns exploration of the optimal allocation of resources between information and management [62, 63]. Finally, it is clear that those maintaining all four knowledge products should invest in accurate documentation of income and expenditure, as well as contributed volunteer time, including the inevitable uncertainties in estimates.

Cost-effectiveness of knowledge products could probably be improved by enhancing the integration of their technological platforms and databases, as they are currently managed relatively independently from each other [7]. Better integrating knowledge products into a spatially explicit platform in the Integrated Biodiversity Assessment Tool would also allow users to explore more easily information from different sources available for their region of interest [57]. Likewise, the costs of managing datasets that are built on a common platform may potentially be lower than managing them independently.

### Costs of knowledge products in the wider context

The study did not systematically compare the costs of knowledge products with other societal investments. This would be an important consideration for future studies as it will inform further decisions on allocation of investments. Nevertheless, the costs of developing and maintaining these knowledge products are very low compared with similar knowledge-generation processes important for humanity. For example, the cost of completing the 2010 US Census was around US$13bn [64], while observations and infrastructure for the Global Observing System for Climate in support of the United Nations Framework Convention on Climate Change (UNFCCC) cost between US$5-7bn in 2010 [65]. These processes, however, include the cost of collecting primary data which this study did not consider. Nevertheless, even if only 10% (e.g., 10% of 5 billion is 500 million) of these totals were invested in collating, interpreting, and publishing secondary data, and 90% in collection of primary data, they dwarf the sums invested in the biodiversity and conservation knowledge products discussed here. It is clear that the development and maintenance of biodiversity knowledge products does not represent a disproportionate economic cost, especially considering their global relevance to inform decisions relating international conventions and agreements, notably the CBD Aichi Biodiversity Targets and the Sustainable Development Goals. It is therefore surprising that none of these knowledge products has long-term sustainable funding models and all are financed in an *ad hoc* fashion, leaving them critically underfunded and at risk of becoming inaccurate, outdated or unavailable. Acknowledging their value, the IUCN Council recently recognized the data underlying the knowledge products as global public goods, and requested IUCN to engage with international finance organizations and interested governments regarding the need to invest in their development and maintenance ([66] pp. 24). This is perhaps a first step to work towards long-term sustainability. At least 4.5 million unique visitors (albeit ignoring potential overlaps between knowledge products) access these knowledge products online each year to visualise information or download tabular or spatial data (See S5 Table), further highlighting the importance of making sure these resources are updated, accurate and relevant to users.

### Conclusion

This study provides the first comprehensive assessment on the costs of developing and maintaining global biodiversity and conservation knowledge through four widely used knowledge products. Understanding the costs of data generation is fundamentally important to ensure the long term sustainability of global biodiversity information. Without data we cannot generate information and build knowledge to make informed decisions or develop indicators to track progress towards biodiversity goals and targets. The people, structures, and processes that make this possible have a financial cost that is often taken for granted by those who benefit from such resources. Generation of authoritative biodiversity and conservation information through knowledge products is affordable if considered at a global scale and offers a good value for money compared with other similar processes. Given the relevance of these knowledge products, we argue that, as recognised by the IUCN Council, they should be regarded as global public goods, fundamental to improved development planning, and sustainable long-term funding mechanisms should be ensured for the sake of nature and humanity.

## Supporting Information

S1 Table Currency conversions and accounting for inflation. Costs were provided in British Pounds (GBP), US Dollars (US$), Euros (EUR), Australian Dollars (AUD), and Swiss Francs (CHF) of the year when the investment took place. We accounted for inflation by calculating the value of the currency in 2014 and then we transformed all currencies to US Dollars (US$).
(DOCX)

S2 Table Calculation *of the cost of achieving pre-defined baselines by 2020*. All extrapolations were derived through data from the sample compiled in the study and projections provided by each knowledge product team.
(DOCX)

S3 Table Summary of data collection for all four knowledge products. The table summarises which costs were collected for each of the four knowledge products and how much of the total number of assesments, available in December 2013, these represent. In cases where 100% of the costs were not collected, the total sum for each knowledge product was increased propotionally to reach 100%.
(DOCX)

S4 Table Extrapolated one-off costs costs per funding source between 1979 and 2013 for all four knowledge products. Extrapolated one-off costs costs per cost categories between 1979 and 2013 for all four knowledge products.
(DOCX)

S5 Table Unique visitors per year to knowledge products’ websites. (DOCX)
