# Supplementary material for: Assessing the Cost of Global Biodiversity and Conservation Knowledge
Source: PLoS One. 2016 Aug 16;11(8):e0160640. doi: 10.1371/journal.pone.0160640 (PMC4986939; doi:10.1371/journal.pone.0160640)
Supplement: S1 Table — Costs were provided in British Pounds (GBP), US Dollars (US$), Euros (EUR), Australian Dollars (AUD), and Swiss Francs (CHF) of the year when the investment took place. We accounted for inflation by calculating the value of the currency in 2014 and then we transformed all currencies to US Dollars (US$). (DOCX) [file pone.0160640.s001.docx]

Supporting Information

S1- Currency conversions and accounting for inflation

Costs were provided in British Pounds (GBP), US Dollars (US$), Euros (EUR), Australian Dollars (AUD), and Swiss Francs (CHF) of the year when the investment took place. We accounted for inflation by calculating the value of the currency in 2014 and then we transformed all currencies to US Dollars (US$).

To account for inflation in Pound sterling we used: <http://www.bankofengland.co.uk/education/Pages/inflation/calculator/flash/default.aspx>

To account for inflation in US dollars we used: <http://www.bls.gov/data/inflation_calculator.htm>

To account for inflation in Euros we used: <http://www.lawyerdb.de/Inflationrate.aspx>

To account for inflation in Australian dollars we used: <http://www.rba.gov.au/calculator/annualDecimal.html>
