# Supplementary material for: Assessing the Cost of Global Biodiversity and Conservation Knowledge
Source: PLoS One. 2016 Aug 16;11(8):e0160640. doi: 10.1371/journal.pone.0160640 (PMC4986939; doi:10.1371/journal.pone.0160640)
Supplement: S2 Table — All extrapolations were derived through data from the sample compiled in the study and projections provided by each knowledge product team. (DOCX) [file pone.0160640.s002.docx]

S2 – Calculation of the cost of achieving pre-defined baselines by 2020. All extrapolations we derived through data from the sample compiled in the study and projections provided by each knowledge product team.

| *Knowledge product* | *Methodology* |
| --- | --- |
| The IUCN Red List of Threatened Species | It includes: 1) annual and one-off costs to upgrade current infrastructure to manage 160,000 species assessments including predicted staff costs; 2) costs of assessing additional species to reach 160,000 based on an average costs of a species assessment until 2013 of US$189 per species (extracted from the database compiled in this study); 3) costs of the Red List Committee and related governance and technical bodies; 4) projected costs of Red List training by 2020; 5) annual costs of other Red List processes. Inflation rate at 2% per year. |
| *Protected Planet* | It includes: 1) annual cost of the WDPA unit considering incremental cost in Convention on Biological Diversity Conferences of the Parties and/or IUCN World Conservation Congress years; 2) cost of implementing the Protected Planet strategy from the strategy provisional budget. Inflation rate at 2% per year. |
| *The World Database of Key Biodiversity Areas* | It includes: 1) annual cost of maintaining the World Bird and Biodiversity Database (WBDB) in which KBA data are managed; 2) cost of identifying additional KBAs to reach 18,088 KBAs (increase existing number of IBAs by 23%); 3) cost of documenting existing KBAs for a wider range of taxa and applying new KBA criteria based on the costs of identifying KBAs for multiple taxa under the CEPF process (see CEPF, 2007); 4) predicted annual cost for governance structures for the identification and documentation of KBAs under the new KBA Standard (IUCN 2014b). Inflation rate at 2% per year. |
| *IUCN Red List of Ecosystems* | It includes: 1) one–one off costs for concept development, data development, training, and infrastructure for a global IUCN Red List of Ecosystems; 2) one-off costs for developing and applying criteria for a first iteration red list; 3) annual cost to maintain data management, assessment unit, and governance structures. Inflation rate at 2% per year. |
