# Supplementary material for: Assessing the Cost of Global Biodiversity and Conservation Knowledge
Source: PLoS One. 2016 Aug 16;11(8):e0160640. doi: 10.1371/journal.pone.0160640 (PMC4986939; doi:10.1371/journal.pone.0160640)
Supplement: S3 Table — The table summarises which costs were collected for each of the four knowledge products and how much of the total number of assesments, available in December 2013, these represent. In cases where 100% of the costs were not collected, the total sum for each knowledge product was increased propotionally to reach 100%. (DOCX) [file pone.0160640.s003.docx]

S3 –Summary of data collection for all four knowledge products. The table summarises which costs were collected for each of the four knowledge products and how much of the total number of assesments, available in December 2013, these represent. In cases where 100% of the costs were not collected, the total sum for each knowledge product was increased propotionally to reach 100%.

| *Knowledge product* | *Organizations that provided data for this study* | *Units* | *Subset* | *Number of units* | *Period covered* | *Percentage of dataset (December 2013)* |
| --- | --- | --- | --- | --- | --- | --- |
| The IUCN Red List of Threatened Species^1^ | IUCN, BirdLife International, University of Rome, Zoological Society of London, NatureServe, Royal BotanicalGardens Kew, and Natural History Museum London. | Species assessments and associated spatial and tabular data | Birds  Marine Species  Cacti  Amphibians  Mammals  Small selection of Reptiles  Freshwater Species  Sampled Red List Species  Red List Unit  Red List Committee and related bodies | 46,492  908  1,480  5,743  5,488  3,612  2,500  14,379  Not applicable  Not applicable | 1985-2013  2008-2013  2008-2012  2001-2004  1996-2013  2005-2012  2009-2012  2002-2013  1999-2013  1999-2013 |  |
| Total |  |  |  | **76,068** |  | **67.73%** |
| Protected Planet^2^ | UNEP-World Conservation Monitoring Centre and IUCN | Protected areas spatial and tabular data | Not applicable | 214,000 | 1981-2013 |  |
| Total |  |  |  | **214,000** |  | **100%** |

| *Knowledge product* | *Organizations that provided data for this study* | *Units* | *Subset* | *Number of units* | *Period covered* | *Percentage of dataset (December 2013)* |
| --- | --- | --- | --- | --- | --- | --- |
| The World Database of Key Biodiversity Areas (KBAs)^3^ | BirdLife International, Alliance for Zero Extinction, IUCN, and CEPF. | The World Database of Key Biodiversity Areas spatial and tabular data | Important Bird and Biodiversity Areas  Alliance for Zero Extinction sites  CEPF KBAs  Freshwater KBAs | **12,300**  **587**  **1,625**  **225** | 1979-2013  2010  2004-2012  2012-2013 |  |
| Total |  |  |  | **17,732** |  | **88.37%** |
| IUCN Red List of Ecosystems^4^ | IUCN, Provita, and University of New South Wales, Australia | Ecosystem assessments | Senegal  Venezuela  Morocco  Continental Americas  Costa Rica  Colombia | 19  18  79  500 | 2004-2013 |  |
| **Total** |  |  |  | **616** |  | **100%** |

***^1^***The dataset assessed was all species published on [www.iucnredlist.org](http://www.iucnredlist.org/) by the end of 2013 including re-assessments. ^2^The dataset assessed was the December 2014 version of World Database on Protected Areas (IUCN and UNEP-WCMC 2014). ***^3^***The dataset assessed was the World Birds and Biodiversity Database.^4^The dataset assessed was all ecosystems assessments completed or about to be completed by end of 2013.
