# Supplementary material for: Assessing the Cost of Global Biodiversity and Conservation Knowledge
Source: PLoS One. 2016 Aug 16;11(8):e0160640. doi: 10.1371/journal.pone.0160640 (PMC4986939; doi:10.1371/journal.pone.0160640)
Supplement: S4 Table — Extrapolated one-off costs costs per cost categories between 1979 and 2013 for all four knowledge products. (DOCX) [file pone.0160640.s004.docx]

S4 –Full list of extrapolated one-off costs between 1979 and 2013 for all four knowledge products per funding sources and cost categories

**Table S4.1 Costs per funding sources for all four knowledge products**

| **FUNDING SOURCES** | **US$ 2014 min.** | | **US$ 2014 mid- point** | | **US$ 2014 max.** | | | | |
| --- | --- | --- | --- | --- | --- | --- | --- | --- | --- |
| **The IUCN Red List of Threatened Species** | **34,075,981** | | **34,935,095** | | **35,794,208** | | | | |
| Philanthropy | 14,220,360 | | 14,243,174 | | 14,265,988 | | | | |
| Governments | 10,259,483 | | 10,259,483 | | 10,259,483 | | | | |
| Non-Governmental Organisation (NGOs) | 5,638,075 | | 5,980,883 | | 6,323,691 | | | | |
| Private Sector | 895,130 | | 895,130 | | 895,130 | | | | |
| No data | 2,749,695 | | 3,243,187 | | 3,736,679 | | | | |
| Multilateral Donor | 313,235 | | 313,235 | | 313,235 | | | | |
| **Protected** **Planet** | **19,055,847** | | **19,055,847** | | **19,055,847** | | | | |
| Philanthropy | 30,636 | | 30,636 | | 30,636 | | | | |
| Governments | 1,476,146 | | 1,476,146 | | 1,476,146 | | | | |
| Non-Governmental Organisation (NGOs) | 727,228 | | 727,228 | | 727,228 | | | | |
| Intergovernmental Organisations (IGOs) | 12,418,825 | | 12,418,825 | | 12,418,825 | | | | |
| Private Sector | 4,403,013 | | 4,403,013 | | 4,403,013 | | | | |
| **The World Database of Key Biodiversity Areas** | **56,250,743** | | **99,106,414** | | **141,962,085** | | | | |
| Philanthropy | 44,447,934 | | 65,720,140 | | 86,992,346 | | | | |
| Governments | 5,219,876 | | 26,480,767 | | 47,741,657 | | | | |
| Non-Governmental Organisation (NGOs) | 3,558,751 | | 3,881,325 | | 4,203,898 | | | | |
| Private Sector | 1,394,974 | | 1,394,974 | | 1,394,974 | | | | |
| Multilateral Donor | 1,615,753 | | 1,615,753 | | 1,615,753 | | | | |
| Financial Institutions | 13,456 | | 13,456 | | 13,456 | | | | |
| **IUCN Red List of Ecosystems** | **6,706,400** | | **6,706,400** | | **6,706,400** | | | | |
| Philanthropy | 4,336,267 | | 4,336,267 | | 4,336,267 | | | | |
| Governments | 161,338 | | 161,338 | | 161,338 | | | | |
| Non-Governmental Organisation (NGOs) | 2,134,445 | | 2,134,445 | | 2,134,445 | | | | |
| No data | 74,350 | | 74,350 | | 74,350 | | | | |
| **TOTAL** | **116,088,971** | | **159,803,756** | | **203,518,540** | | | | |
| Table S4.2 Costs per cost categories for all four knowledge products | | | | | | | | | |
| **COST CATEGORIES** | | **US$ 2014 min.** | | **US$ 2014 mid-point** | | **US$ 2014 max.** | **Volunteer time min. (days)** | **Volunteer time mid-point (days)** | **Volunteer time max. (days)** |
| **IUCN Red List of Threatened Species** | | **34,075,981** | | **34,935,095** | | **35,794,208** | **46,495** | **50,110** | **53,726** |
| Infrastructure | | 2,377,932 | | 2,384,032 | | 2,390,132 | - | - | - |
| Personnel costs | | 23,820,171 | | 24,170,140 | | 24,520,109 | 40,285 | 43,901 | 47,516 |
| Travel and workshops | | 4,338,309 | | 4,347,861 | | 4,357,414 | 6,210 | 6,210 | 6,210 |
| Publications & outreach | | 835,514 | | 835,514 | | 835,514 | 835,514 | 835,514 | 835,514 |
| No data | | 2,704,056 | | 3,197,548 | | 3,691,040 | 2,704,056 | 3,197,548 | 3,691,040 |
| **Protected Planet** | | **19,055,847** | | **19,055,847** | | **19,055,847** | **1,190** | **1,190** | **1,190** |
| Infrastructure | | 1,876,879 | | 1,876,879 | | 1,876,879 | - | - | 1,190 |
| Personnel costs | | 13,910,092 | | 13,910,092 | | 13,910,092 | 1,190 | 1,190 | - |
| Travel and workshops | | 979,217 | | 979,217 | | 979,217 | - | - | - |
| Publications & outreach | | 2,289,659 | | 2,289,659 | | 2,289,659 | - | - |  |
| **The World Database of Key Biodiversity Areas** | | **56,250,743** | | **99,106,414** | | **141,962,085** | **18,654** | **18,654** | **18,654** |
| Infrastructure | | 535,892 | | 600,407 | | 664,921 | 68 | 68 | 68 |
| Personnel costs | | 40,008,739 | | 70,032,045 | | 100,055,351 | 18,389 | 18,389 | 18,389 |
| Travel and workshops | | 10,611,071 | | 19,115,428 | | 27,619,784 | 95 | 95 | 95 |
| Publications & outreach | | 5,095,041 | | 9,358,534 | | 13,622,028 | 102 | 102 | 102 |
| **IUCN Red List of Ecosystems** | | **6,706,400** | | **6,706,400** | | **6,706,400** | **397** | **397** | **397** |
| Infrastructure | | 166,204 | | 166,204 | | 166,204 | - | - | - |
| Personnel costs | | 5,664,886 | | 5,664,886 | | 5,664,886 | 397 | 397 | 397 |
| Travel and workshops | | 722,631 | | 722,631 | | 722,631 | - | - | - |
| Publications & outreach | | 152,680 | | 152,680 | | 152,680 | - | - | - |
| TOTAL | | **116,088,971** | | **159,803,756** | | **203,518,540** | **66,735** | **70,351** | **73,967** |
