# Supplementary material for: Assessing the Cost of Global Biodiversity and Conservation Knowledge
Source: PLoS One. 2016 Aug 16;11(8):e0160640. doi: 10.1371/journal.pone.0160640 (PMC4986939; doi:10.1371/journal.pone.0160640)
Supplement: S5 Table — (DOCX) [file pone.0160640.s005.docx]

S5 – Unique visitors per year to knowledge products’ websites.

| *Knowledge product* | *Unique visitors per year* | *Method* |
| --- | --- | --- |
| The IUCN Red List of Threatened Species | 4,222,660 | Based on google analytics on [www.iucnredlist.org](http://www.iucnredlist.org/) for 2014 (3,573,721) plus [www.birdlife.org/datazone/species](http://www.birdlife.org/datazone/species) for 2014 (648,039). |
| Protected Planet | 196,376 | Based on google analytics on [www.protectedplanet.net](http://www.protectedplanet.net/) for 2014. |
| The World Database of Key Biodiversity Areas | 95,549 | Based on google analytics on [www.birdlife.org/datazone/sites](http://www.birdlife.org/datazone/sites) for 2014. |
| IUCN Red List of Ecosystems | 8,138 | Unique visitors to [www.iucnredlistofecosystems.org](http://www.iucnredlistofecosystems.org/) in 2014. |
| *Total* | ***4,522,723*** |  |
